# Integrative analysis of causal associations between neurodegenerative diseases and colorectal cancer

**DOI:** 10.1016/j.heliyon.2024.e35432

**Published:** 2024-07-30

**Authors:** Feifan Wang, Lu Chen, Mengke Nie, Zhongxin Li

**Affiliations:** a Gastrointestinal Disease Diagnosis and Treatment Center, The First Hospital of Hebei Medical University, Shijiazhuang, 050000, China; b Department of Medical Oncology and Radiation Sickness, Peking University Third Hospital, Beijing, 100191, China; c Department of General Practice, Huaihe Hospital of Henan University, Kaifeng, 475000, China

**Keywords:** Colorectal cancer, Neurodegenerative disease, Mendelian randomization, Alzheimer's disease, Lewy body dementia, Colocalization analysis

## Abstract

**Background:**

Observational studies have shown that the correlation between neurodegenerative diseases and colorectal cancer (CRC) remains controversial. Therefore, this study aimed to verify the causal association between these two diseases.

**Methods:**

Mendelian randomization (MR) analysis was used to assess the causal relationships between five major neurodegenerative diseases and CRC. Multivariable MR (MVMR) analysis was conducted to assess the direct causal effect of neurodegenerative diseases on CRC. Colocalization and pathway enrichment analyses were conducted to further elucidate our results. Sensitivity analysis was conducted to assess the robustness of the results.

**Results:**

Genetically predicted Alzheimer's disease (AD) nominally increased CRC risk (OR = 1.0620, 95%CI = 1.0127–1.1136, *P* = 0.013). There was no causal effect of genetically predicted CRC on neurodegenerative diseases. Furthermore, we demonstrated that genetically predicted AD marginally increased colon cancer risk (OR = 1.1621, 95%CI = 1.0267–1.3153, *P* = 0.017). Genetically predicted Lewy body dementia (LBD) had a significant causal effect on the increasing risk of colon cancer (IVW OR = 1.1779, 95%CI = 1.0694–1.2975, *P* = 0.001). MVMR indicated that effect of AD on colon cancer was driven by LBD, type 2 diabetes, body mass index, low-density lipoprotein cholesterol, high-density lipoprotein cholesterol, triglyceride, total cholesterol (TC), processed meat consumption, smoking, alcohol consumption, and educational attainment, whereas the effect of LBD on colon cancer was only influenced by TC. Colocalization and pathway enrichment analysis suggested that LBD and colon cancer possibly shared causal variants (nearby gene APOE), and ERBB4 signaling and lipid metabolism may mediate the causal association between LBD and colon cancer. Sensitivity analysis confirmed the reliability of our findings.

**Conclusions:**

Our study demonstrated that genetic vulnerabilities to AD nominally increased the overall risk of CRC and colon cancer. Genetically predicted LBD indicated an elevated risk of colon cancer, potentially linked to ERBB4 signaling and lipid metabolism.

## Highlights

• Genetic vulnerabilities to AD nominally increased the risk of overall CRC and colon cancer.
• Genetically predicted LBD had a significant causal effect on the increasing risk of colon cancer.
• AD’s effect on colon cancer was driven by the common risk factors, while LBD’s effect was only driven by blood lipid.
• ERBB4 signaling and lipid metabolism might mediate the causal association between LBD and colon cancer.

## 1 Introduction

Neurodegenerative diseases and colorectal cancer (CRC) are chronic and progressive diseases, for which aging is a significant risk factor [1]. The incidence of these diseases is gradually increasing with the acceleration of population aging, which has a considerable socioeconomic impact [2,3]. Therefore, greater attention to these two diseases is imperative. Dysfunction, slow progressive loss of neurons in the central nervous system, impairment of synaptic plasticity, progressive muscle atrophy or wasting, and proteinopathies, such as misfolded amyloid-β (Aβ) and tau in Alzheimer's disease (AD), α-synuclein in Parkinson's disease (PD), are all characteristics of neurodegenerative diseases. These pathological processes eventually impair memory, movement, and cognition [4]. CRC is the second major cause of cancer-related deaths and the third most common cancer globally. Therefore, it is crucial to investigate the risk factors and pathophysiology of CRC for better prevention and treatment [2]. Similar to other cancers, CRC is associated with many pathological processes, including excess cell proliferation, growth factor receptor overactivation, resistance to cell death, induction of angiogenesis, metastasis, and immune evasion [5].

Neurodegenerative diseases and cancer exhibit opposing mechanisms, involving progressive cell death and uncontrolled cell proliferation. Observational studies have shown an inverse correlation between neurodegenerative diseases and CRC [6,7]. Further research has identified consistent pathophysiological mechanisms in these two diseases, including mitochondrial dysfunction, autophagy, DNA damage, and oxidative stress [8]. In addition, there is increasing evidence that the brain-gut axis plays a crucial role in connecting neurological and gastrointestinal disorders. Therefore, the association between neurodegenerative diseases and CRC has shifted from a negative correlation in the past to a positive correlation between certain neurodegenerative diseases and CRC at present, certain observational studies support this standpoint [9,10]. Unfortunately, because neurodegenerative diseases usually persist for over a decade, the actual onset of neurodegeneration might precede clinical manifestations by many years. This makes it difficult for observational studies to determine the precise onset times. Furthermore, various measurement errors, potential biases, and confounding factors (e.g., disease development and treatment) in the observational studies may have obscured the actual causal association.

Mendelian randomization (MR) analysis can eliminate this bias in observational studies. Genetic variation is used as an instrument variable (IV) to deduce the causal association between exposure and outcome in the MR analysis. First, confounding factors can theoretically be avoided in MR analysis. Second, exposure is associated with IV, which appears before the outcome, thus excluding the influence of reverse causation [11].

In this study, we performed MR analysis to evaluate the causal relationship between five major neurodegenerative diseases, including AD, PD, amyotrophic lateral sclerosis (ALS), Lewy body dementia (LBD), frontotemporal dementia (FTD), and CRC (including colon cancer and rectum cancer). Multivariable MR (MVMR) analysis was conducted to assess the direct causal effect of neurodegenerative diseases on CRC. Finally, colocalization and pathway enrichment analyses were performed to further elucidate the results.

## 2 Materials and Methods

### 2.1 Study design

**Fig. 1:**
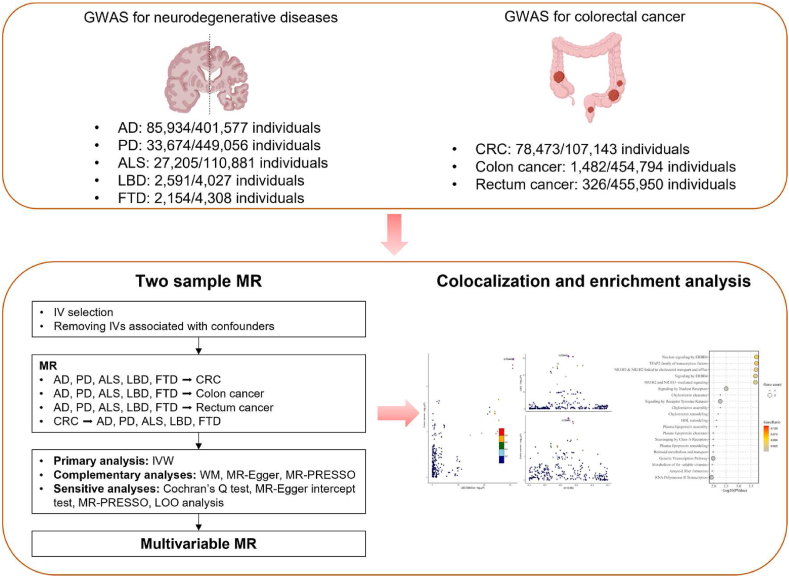
Overview of our study design. AD, Alzheimer's disease; PD, Parkinson's disease; ALS, amyotrophic lateral sclerosis; LBD, Lewy body dementia; FTD, frontotemporal dementia; CRC, colorectal cancer.

We displayed the overview of our study in Fig. 1. In general, there are three assumptions needed to be satisfied in the bidirectional MR analysis: (1) IVs for AD, PD, ALS, LBD, FTD, and CRC are closely linked to exposure; (2) IVs are not related to confounders in the exposure-outcome association; (3) IVs directly through exposure influence the outcome instead of through other pathways.

### 2.2 Data sources

Genetic association data of AD (case = 85,934, controls = 401,577, N = 487,511), PD (case = 33,674, controls = 449,056, N = 482,730), ALS (case = 27,205, controls = 110,881, N = 138,086), LBD (case = 2,591, controls = 4,027, N = 6618), FTD (case = 2,154, controls = 4,308, N = 6462), CRC (case = 78,473, controls = 107,143, N = 185,616), body mass index (BMI) (N = 681,275), smoking (cigarettes per day, N = 377,334), alcohol consumption (drinks per week, N = 941,280), and educational attainment (N = 405,072) were obtained from large-scale genome-wide association studies (GWAS) [[12], [13], [14], [15], [16], [17], [18], [19], [20]]. Summary data of GWAS for colon cancer (case = 1,482, controls = 454,794, N = 456,276), rectum cancer (case = 326, controls = 455,950, N = 456,276), total cholesterol (TC) (N = 437,878), and processed meat consumption (N = 448,303) were from the UK Biobank study [21]. Genetic association data of low-density lipoprotein cholesterol (LDL-C) (N = 343,621), high-density lipoprotein cholesterol (HDL-C) (N = 315,133), triglyceride (TG) (N = 343,992), and type 2 diabetes (T2D) (N = 462,933) were from the IEU Open GWAS database (https://gwas.mrcieu.ac.uk/). The detailed data sources are listed in Table S1.

### 2.3 Instrumental variables selection

We confirmed single nucleotide polymorphisms (SNPs) that were associated with genome-wide significance (*P* < 5 × 10^−8^) associations for exposure and performed linkage disequilibrium (LD) clumping by setting r^2^＜0.001 and clump distance＞10,000 kb to ensure the SNPs are independent and satisfy the first assumption. Because of only one IV for FTD, we used a more relaxed significance threshold (*P* < 1 × 10^−5^). In addition, to prevent weak IVs, we calculated R^2^ (R^2^ = 2 × EAF × (1−EAF) × β^2^), representing the proportion of variation explained by IVs, and the *F*-statistic (*F* = R^2^ (n-k-1)/k (1-R^2^)), indicating the strong association between IVs and exposure. We identified 60 SNPs for AD, 23 for PD, 12 for ALS, 5 for LBD, 13 for FTD, and 116 for CRC. The details of IVs for exposures are shown in Tables S2–S7.

### 2.4 Mendelian randomization analysis

Four common MR approaches, including inverse variance weighted (IVW), weighted median (WM), MR-Egger, and MR-Pleiotropy Residual Sum and Outlier (MR-PRESSO) methods, were utilized to evaluate causal associations between five neurodegenerative diseases and CRC, including colon cancer and rectum cancer. IVW is the main evaluation method because it has the most significant statistical power and can identify reliable causal estimates without directional pleiotropy [22]. Because MR-Egger and WM methods could provide more robust but wider confidence interval estimations in broader conditions, we used them to complement IVW estimations.

To eliminate the influence of confounders, we used two different methods. In the first one, we searched for pleiotropic SNPs in PhenoScanner (http://www.phenoscanner.medschl.cam.ac.uk/). Then, we excluded IVs significantly associated (*P* < 1 × 10^−5^) with other traits, including BMI, T2D, smoking, alcohol consumption, processed meat consumption, inflammatory bowel disease, and cholesterol, which could affect our results and used the remaining IVs for MR analysis. In the second one, we conducted MVMR analysis with adjustment for genetically predicted other identified neurodegenerative diseases, T2D, BMI, LDL-C, HDL-C, TG, TC, processed meat consumption, smoking, alcohol consumption, and educational attainment, which have been demonstrated to be associated with CRC [[23], [24], [25], [26]].

Furthermore, we conducted a comprehensive sensitivity analysis to detect potential violations of the model assumptions in the MR analysis. We used Cochran's Q test to evaluate the heterogeneity among IVs (value of *P* < 0.05 suggesting heterogeneity). The MR-PRESSO method was also utilized to detect outlier IVs and correct horizontal pleiotropy. We conducted the MR-Egger intercept test to detect the directional pleiotropy of IVs (value of *P* < 0.05 suggesting pleiotropy) [27]. We also performed the Leave-one-out analysis to evaluate the reliability of the results of the MR analysis.

All MR analyses were conducted by the “TwoSampleMR” R package, the “MendelianRandomization” R package and the “MRPRESSO” R package in R software (version 4.2.2). Multiple comparisons were made by Bonferroni correction (*P* < 0.05/5), which was regarded as evidence of statistical significance. However, *P* < 0.05 was assumed nominally significant of a causal association.

### 2.5 Colocalization and enrichment analysis

For neurodegenerative diseases that appeared causally associated with CRC risk in the MR analysis, we performed colocalization analysis using the “coloc” R package to evaluate the probability of shared causal variants between them. Colocalization analysis was performed by generating ±50 kb windows from the top SNP of neurodegenerative diseases. We obtained posterior probability for 5 hypotheses (H0–H4) in a Bayesian framework [28]. PP.H4 ≥ 80 % of the colocalization analysis (H4) indicated strong-evidence for shared causal variants. Medium-strength evidence for shared causal variants was defined as 50 % < PP.H4 < 80 % [29]. Finally, we conducted the pathway enrichment analysis to identify overrepresented pathways in the top SNP utilizing Reactome [30]. The results were adjusted for false discovery rate.

## 3 Results

### 3.1 Effect of neurodegenerative diseases on CRC

**Fig. 2:**
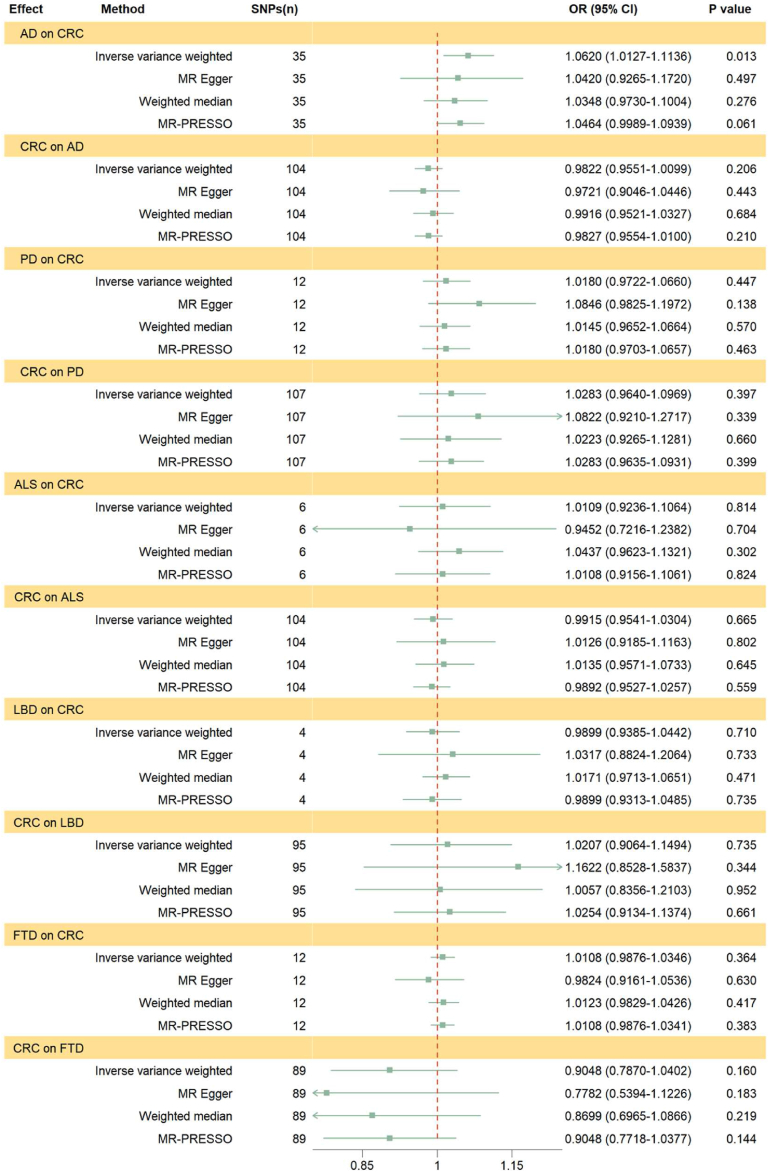
Forest plot for results of the bidirectional Mendelian randomization analysis. AD, Alzheimer's disease; PD, Parkinson's disease; ALS, amyotrophic lateral sclerosis; LBD, Lewy body dementia; FTD, frontotemporal dementia; CRC, colorectal cancer.

After excluding potential pleiotropic and palindromic SNPs, we used the remaining SNPs to evaluate the effect of neurodegenerative diseases on CRC. Based on Bonferroni correction, we found that genetically predicted AD could nominally raise the risk of CRC using the IVW method (IVW OR = 1.0620, 95%CI = 1.0127–1.1136, *P* = 0.013). Moreover, the consistent impact direction was shown in other methods (Fig. 2). The MR-PRESSO global test showed no horizontal pleiotropy (*P* = 0.051). The MR analysis showed no effect of PD on CRC. However, the MR-PRESSO global test showed horizontal pleiotropy (*P* = 0.013). After one outlier was removed (rs6741007), the result of the IVW method was still the same as before (IVW *P* = 0.447) (Fig. 2). There was no causal effect of genetically predicted ALS on CRC in all MR methods. However, the MR-PRESSO global test showed horizontal pleiotropy (*P* = 0.006) and found one outlier (rs517339). After the outlier was removed, the results of the MR analysis were not changed (IVW *P* = 0.814) (Fig. 2). There was no causal association between LBD and CRC in all MR methods (Fig. 2). Furthermore, the MR-PRESSO global test showed no horizontal pleiotropy (*P* = 0.116). The MR analysis showed no effect of FTD on CRC. However, the MR-PRESSO global test showed horizontal pleiotropy (*P* < 0.001). After one outlier was removed (rs9268877), the result of the IVW method was not changed (IVW *P* = 0.364) (Fig. 2).

### 3.2 Effect of CRC on neurodegenerative diseases

After excluding potential pleiotropic and palindromic SNPs, we used the remaining SNPs to evaluate the effect of CRC on neurodegenerative diseases. We found no effect of CRC on AD using the MR analysis. However, the MR-PRESSO global test showed horizontal pleiotropy (*P* < 0.001). After two outliers were removed (rs2527927 and rs11692435), the results of MR analysis were consistent with before (IVW *P* = 0.206) (Fig. 2). We did not find that a genetic predisposition to CRC was associated with PD. However, the MR-PRESSO global test showed horizontal pleiotropy (*P* = 0.010). After one outlier was removed (rs12078075), the results of the MR analysis were not changed (IVW *P* = 0.397, MR-Egger *P* = 0.339, WM *P* = 0.660, MR-PRESSO *P* = 0.399) (Fig. 2). In addition, MR analysis showed no effect of CRC on ALS (IVW *P* = 0.665) (Fig. 2). The MR-PRESSO global test showed no horizontal pleiotropy (*P* = 0.107). There was also no causal association between CRC and LBD in all MR methods (Fig. 2). The MR-PRESSO global test showed no horizontal pleiotropy (*P* = 0.116). We found no effect of CRC on FTD using the MR analysis (IVW *P* = 0.160) (Fig. 2). Moreover, the MR-PRESSO global test showed no horizontal pleiotropy (*P* = 0.740).

### 3.3 Sensitivity analysis

We used several sensitivity analyses to evaluate the reliability of our MR analysis. The Cochran's Q test showed slight heterogeneity in partial MR estimates, and we used the random effects IVW method to alleviate the problem (Table S8). The MR-Egger intercept test indicated no directional pleiotropy in all MR estimates (Table S8). Forest and funnel plots among all IVs in the bidirectional MR analysis are shown in Figs. S1–S5. The plots of the leave-one-out sensitivity analysis demonstrated that no SNP affected the overall results in our bidirectional MR analysis (Figs. S1–S5).

### 3.4 Effect of neurodegenerative diseases on colon cancer and rectum cancer

We further explored the causal association between neurodegenerative diseases and the site of CRC. After Bonferroni correction for multiple tests, we found that genetically predicted AD nominally increased the risk of colon cancer (IVW OR = 1.1621, 95%CI = 1.0267–1.3153, *P* = 0.017) (Table 1). LBD and colon cancer showed a significant causal association. we found that genetically predicted LBD increased the risk of colon cancer (IVW OR = 1.1779, 95%CI = 1.0694–1.2975, *P* = 0.001) (Table 1). However, we found no significant causal association between the other three neurodegenerative diseases and colon cancer (Table 1). The Cochran's Q test indicated no heterogeneity, and the MR-Egger intercept and MR-PRESSO global tests also showed no significant horizontal pleiotropy in all MR estimates (Table S9).

**Table 1:** Results of the MR analysis between neurodegenerative diseases and colon cancer and rectum cancer. AD, Alzheimer's disease; PD, Parkinson's disease; ALS, amyotrophic lateral sclerosis; LBD, Lewy body dementia; FTD, frontotemporal dementia.

Exposure	Outcome	MR method	OR (95 % CI)	*P*
AD	Colon cancer	Inverse variance weighted	1.1621 (1.0267–1.3153)	0.017
		MR-Egger	1.1913 (0.9599–1.4786)	0.118
		Weighted median	1.2439 (1.0312–1.5005)	0.023
		MR-PRESSO	1.1659 (1.0474–1.2844)	0.011
AD	Rectum cancer	Inverse variance weighted	0.7999 (0.6146–1.0409)	0.097
		MR-Egger	1.1591 (0.7322–1.8347)	0.531
		Weighted median	1.0890 (0.7450–1.5917)	0.660
		MR-PRESSO	0.8126 (0.5627–1.0626)	0.104
PD	Colon cancer	Inverse variance weighted	0.9242 (0.8230–1.0379)	0.183
		MR-Egger	1.1043 (0.8233–1.4813)	0.515
		Weighted median	0.9066 (0.7722–1.0644)	0.231
		MR-PRESSO	0.9242 (0.8201–1.0283)	0.152
PD	Rectum cancer	Inverse variance weighted	1.0219 (0.9097–1.1480)	0.715
		MR-Egger	1.0284 (0.7662–1.3803)	0.854
		Weighted median	0.8140 (0.9618–1.1364)	0.647
		MR-PRESSO	1.0219 (0.9183–1.1255)	0.686
ALS	Colon cancer	Inverse variance weighted	0.9247 (0.7271–1.1760)	0.523
		MR-Egger	0.9959 (0.5588–1.7752)	0.989
		Weighted median	1.0932 (0.7764–1.5394)	0.610
		MR-PRESSO	0.9247 (0.6870–1.1624)	0.532
ALS	Rectum cancer	Inverse variance weighted	1.1127 (0.8743–1.4162)	0.385
		MR-Egger	1.3505 (0.7706–2.3670)	0.319
		Weighted median	1.1992 (0.8710–1.6510)	0.266
		MR-PRESSO	1.1127 (0.8890–1.3365)	0.471
LBD	Colon cancer	Inverse variance weighted	1.1779 (1.0694–1.2975)	0.001
		MR-Egger	1.2499 (1.0630–1.4696)	0.007
		Weighted median	1.2051 (1.0794–1.3456)	0.001
		MR-PRESSO	1.1779 (1.1089–1.2469)	0.010
LBD	Rectum cancer	Inverse variance weighted	1.0978 (0.9965–1.2095)	0.059
		MR-Egger	1.2304 (1.0462–1.4471)	0.009
		Weighted median	1.1022 (0.9879–1.2298)	0.081
		MR-PRESSO	1.0978 (1.0066–1.1891)	0.012
FTD	Colon cancer	Inverse variance weighted	0.9322 (0.8349–1.0409)	0.212
		MR-Egger	0.8563 (0.5985–1.2252)	0.414
		Weighted median	0.9119 (0.8039–1.0344)	0.151
		MR-PRESSO	0.9322 (0.8220–1.0425)	0.634
FTD	Rectum cancer	Inverse variance weighted	0.9783 (0.8759–1.0928)	0.698
		MR-Egger	0.8076 (0.5732–1.1381)	0.248
		Weighted median	1.0097 (0.8802–1.1583)	0.890
		MR-PRESSO	0.9784 (0.8677–1.0890)	0.705

The results of the MR analysis showed no significant causal association between neurodegenerative diseases and rectum cancer (Table 1). The Cochran's Q test indicated no heterogeneity, and the MR-Egger intercept and MR-PRESSO global tests also showed no significant horizontal pleiotropy in all MR estimates (Table S9).

Figs. S6–10 show the forest and funnel plots among all IVs in the MR analysis. The plots of the leave-one-out sensitivity analysis demonstrated that our findings were reliable (Figs. S6–10).

### 3.5 MVMR analysis

The MVMR analysis was conducted to assess whether the association between genetically predicted AD and LBD and increased risk of colon cancer was influenced by other neurodegenerative diseases, T2D, BMI, LDL-C, HDL-C, TG, TC, processed meat consumption, smoking, alcohol consumption, and educational attainment. When adjusting for these factors, the effect of genetically predicted AD on colon cancer was attenuated (Table 2). However, the association between genetically predicted LBD and increased risk of colon cancer was only driven by TC (Table 3). The MVMR Egger intercept test showed no horizontal pleiotropy (Table 2, Table 3).

**Table 2:** Results of the MVMR analysis between AD and colon cancer. AD, Alzheimer's disease; LBD, Lewy body dementia; T2D, type 2 diabetes; BMI, body mass index; LDL, low-density lipoprotein cholesterol; HDL, high-density lipoprotein cholesterol; TG, triglyceride; TC, total cholesterol.

Adjustment	OR (95 % CI)	*P*	MVMR Egger		
			Intercept	SE	*P*
LBD	0.9436 (0.8123–1.0750)	0.385	−0.011	0.008	0.200
T2D	0.8869 (0.7184–1.0555)	0.164	−0.009	0.018	0.619
BMI	0.9185 (0.6598–1.1772)	0.521	0.001	0.003	0.768
LDL	0.9792 (0.8361–1.1223)	0.773	−0.008	0.005	0.112
HDL	1.0346 (0.8954–1.1737)	0.628	0.001	0.003	0.765
TG	1.1618 (0.9796–1.3441)	0.105	−0.005	0.006	0.439
TC	0.9474 (0.6991–1.1958)	0.670	0.001	0.005	0.885
Processed meat consumption	0.9901 (0.8613–1.1188)	0.879	−0.003	0.008	0.691
Smoking	0.9871 (0.8361–1.1381)	0.866	−0.005	0.009	0.593
Alcohol consumption	0.9841 (0.8571–1.1112)	0.805	−0.008	0.007	0.222
Educational attainment	0.9753 (0.8350–1.1157)	0.727	−0.006	0.005	0.261

**Table 3:** Results of the MVMR analysis between LBD and colon cancer. AD, Alzheimer's disease; LBD, Lewy body dementia; T2D, type 2 diabetes; BMI, body mass index; LDL, low-density lipoprotein cholesterol; HDL, high-density lipoprotein cholesterol; TG, triglyceride; TC, total cholesterol.

Adjustment	OR (95 % CI)	*P*	MVMR Egger		
			Intercept	SE	*P*
AD	1.1366 (1.0248–1.2483)	0.024	−0.011	0.008	0.200
T2D	1.1780 (1.0610–1.3080)	0.002	−0.034	0.028	0.231
BMI	1.0754 (1.0007–1.1557)	0.048	0.001	0.005	0.865
LDL	1.1140 (1.0102–1.2179)	0.042	0.000	0.006	0.979
HDL	1.0931 (1.0127–1.1734)	0.029	−0.004	0.004	0.305
TG	1.1514 (1.0730–1.2298)	0.000	0.003	0.004	0.388
TC	1.0030 (0.8609–1.1451)	0.967	−0.006	0.005	0.267
Processed meat consumption	1.1901 (1.0940–1.2861)	0.000	0.010	0.012	0.398
Smoking	1.1651 (1.0544–1.2874)	0.003	0.008	0.015	0.610
Alcohol consumption	1.1607 (1.0627–1.2587)	0.003	−0.012	0.009	0.183
Educational attainment	1.1677 (1.0775–1.2578)	0.001	−0.021	0.019	0.251

### 3.6 Colocalization and pathway enrichment analysis

**Fig. 3:**
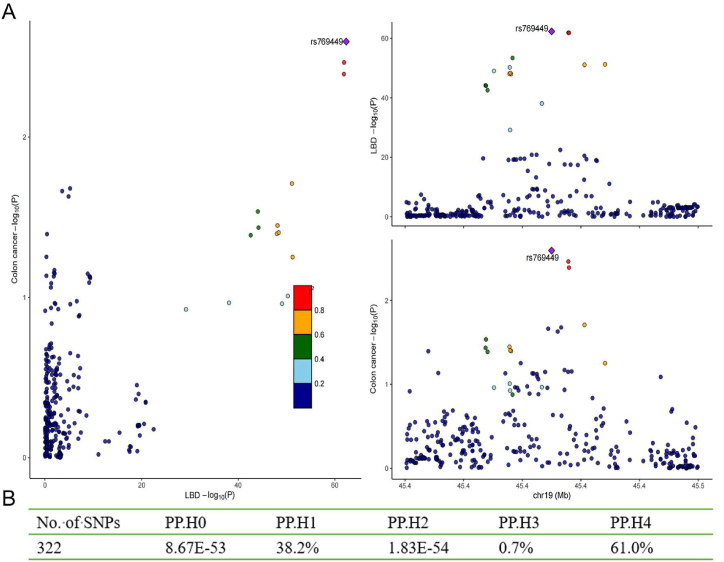
(A) Regional association plots for LBD and colon cancer at the chromosome 19 locus overlapping APOE. (B) Colocalization analysis of LBD and colon cancer. PP.H0 = neither LBD nor colon cancer risk has a genetic association in the region, PP.H1 = only LBD has a genetic association in the region, PP.H2 = only colon cancer risk has a genetic association in the region, PP.H3 = both LBD and colon cancer risk are associated but have different causal variants, PP.H4 = both LBD and colon cancer risk are associated and share a single causal variant.

**Fig. 4:**
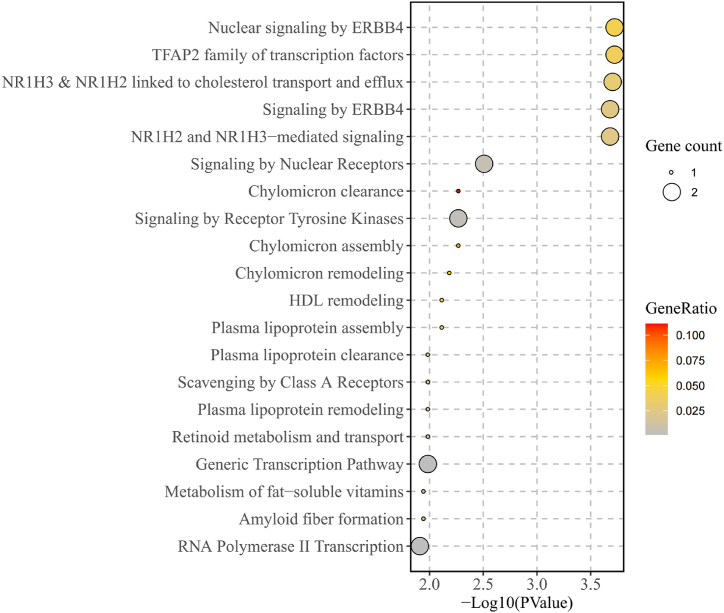
Top 20 significantly enriched pathways in the pathway enrichment analysis.

We performed colocalization analysis to evaluate the probability of shared causal variants between neurodegenerative diseases (AD and LBD) and colon cancer. There was no evidence that AD and colon cancer shared causal variants (PP.H4 = 8.1 %) (Fig. S11). However, there was medium-strength evidence that LBD and colon cancer shared causal variants (PP.H4 = 61.0 %) (Fig. 3B). Regional Manhattan plots examined the association of all SNPs ±50 kb from the top SNP for LBD (rs769449, nearby gene APOE) for their association with LBD and with colon cancer risk (Fig. 3A). Then, we performed pathway enrichment analysis for APOE. The results of enrichment analysis indicated that twenty-eight out of thirty-four pathways were significantly enriched (Table S10). They are closely associated with ERBB4 signaling, NR1H2 and NR1H3-mediated signaling, and lipid metabolism (Fig. 4 and Table S10). It can be inferred that these pathways may mediate the causal association between LBD and colon cancer. Fig. 4 displayed the top 20 significantly enriched pathways in the pathway enrichment analysis.

## 4 Discussion

Using MR analysis, our study provided convincing evidence that genetic vulnerabilities to AD nominally increased the risk of CRC (IVW OR = 1.0620, 95%CI = 1.0127–1.1136, *P* = 0.013). However, there was no causal association between the other four neurodegenerative diseases (PD, ALS, LBD, and FTD) and CRC. We found that CRC had no causal effect on neurodegenerative diseases. Furthermore, genetically predicted AD nominally increased the risk of colon cancer (IVW OR = 1.1621, 95%CI = 1.0267–1.3153, *P* = 0.017). Genetically predicted LBD demonstrated a significant causal effect on the increased risk of colon cancer (IVW OR = 1.1779, 95%CI = 1.0694–1.2975, *P* = 0.001). MVMR analysis confirmed that the effect of AD on colon cancer was partially influenced by LBD, T2D, BMI, LDL-C, HDL-C, TG, TC, processed meat consumption, smoking, alcohol consumption, and educational attainment. However, the association between genetically predicted LBD and an increased risk of colon cancer was only driven by TC. Colocalization analysis suggested that AD and colon cancer shared no causal variants (PP.H4 = 8.1 %), whereas LBD and colon cancer possibly shared causal variants (PP.H4 = 61.0 %) and had one shared locus (rs769449, nearby gene APOE). ERBB4 signaling and lipid metabolism may mediate the causal association between LBD and colon cancer. To the best of our knowledge, this is the first study to construct an integrative analysis to explore causal associations between neurodegenerative diseases and CRC.

Given the increasing incidences of neurodegenerative diseases and CRC, it is necessary to elucidate the relationship between these two diseases. However, the association between these two diseases remains controversial according to current observational studies. MR analysis is not susceptible to confounding factors and reverse causality bias, allowing for more accurate conclusions. We found that genetic vulnerabilities to AD nominally increased the risk of CRC, which is consistent with previous observational research. An observational study revealed that individuals at high risk for AD had a considerably higher incidence of CRC than other populations (RR = 1.30, 95%CI = 1.21–1.39, *P* < 0.001) [10]. It should be noted that the findings of previous studies differ from ours. A cohort study reported contradictory findings, revealing an inverse correlation between AD and CRC [6]. The findings may have resulted from the interaction between genetics and environment, where the non-genetic effect might have had a negative correlation and a greater magnitude than the positively correlated genetic effect. The actual sequence and time of onset of AD and CRC were not available in the observational study because of the long incubation periods of AD and CRC. These factors could be crucial causes for the inverse correlation between AD and CRC risk observed in the aforementioned study. Previous observational studies have revealed that patients with PD and ALS may have a decreased incidence of CRC; nevertheless, no study has explored the association between LBD and CRC [7]. However, our study indicated that the associations observed in previous studies may be related to other factors, including racial differences, confounders, or biases, rather than genetic variants. We found no causal relationship between the other four neurodegenerative diseases (PD, ALS, LBD, and FTD) and CRC, possibly because of differences in the pathogenesis of neurodegenerative diseases. However, the potential mechanisms underlying the association between these neurodegenerative diseases and CRC remain unclear. Moreover, we found no causal effect of genetically predicted CRC on neurodegenerative diseases.

Furthermore, we observed that genetically predicted AD nominally increased the risk for colon cancer. The MVMR analysis further confirmed that the effect of AD on colon cancer was influenced by common risk factors of colon cancer. Colocalization analysis suggested that AD and colon cancer shared no causal variants. The main characteristics of AD pathology are abnormal deposits of Aβ protein and phosphorylated tau in the patients with AD. AD model mice displayed the accumulation of Aβ in enteric neurons, which could lead to neuronal structure damage and a decrease in neuronal numbers. This impairment of intestinal barrier function may contribute to tumorigenesis [31]. BECN1 is critical in regulating autophagy and maintaining cellular homeostasis. The expression of BECN1 was significantly decreased in the brains of patients with AD [32]. Low expression of BECN1, a tumor suppressor, can lead to tumorigenesis [33]. A previous meta-analysis of the gut microbiome dataset of AD patients revealed alterations of significantly lower abundance of short-chain fatty acids producers, including *Firmicutes*, *Clostridiaceae*, *Lachnospiraceae*, and *Rikenellaceae* [34]. The changes in gut microbiota and their Metabolites might promote gut inflammation and injure the intestinal barrier, increasing the risk of colon cancer [35]. Although we found that genetic vulnerabilities to AD could increase the risk of colon cancer, MVMR and colocalization analysis might not support this result. Furthermore, it was undeniable that there were some opposite mechanisms between these two diseases. Research demonstrated that inhibition of Pin 1 and the Wnt signaling pathway, together with upregulation of p53, were found in the development of AD. However, these mechanisms were completely reversed in colon cancer [36]. Therefore, vivo and vitro studies on this topic are still necessary for the future. In general, the underlying mechanisms between AD and CRC are complex and deserve further investigation.

We found that genetically predicted LBD predicted a higher risk of colon cancer. MVMR analysis indicated that the association between genetically predicted LBD and increased risk of colon cancer was only driven by TC. Moreover, colocalization analysis suggested that LBD and colon cancer possibly shared causal variants and had one shared locus (rs769449, nearby gene APOE). Enrichment analysis indicated that ERBB4 signaling and lipid metabolism might mediate the causal association between LBD and colon cancer. The main pathogenesis of LBD is the deposition of α-synuclein in the nervous system and brain [37]. We first reported that genetically predicted LBD could increase the risk of colon cancer. Because there is an overlap in the genetics of LBD and AD, we presume that they may increase the risk of colon cancer through the same mechanism [38]. Both two diseases may lead to the accumulation of misfolded proteins in the gut, which can promote intestinal inflammation and tumorigenesis. Previous studies reported that Biological aggression and a poor prognosis in CRC were linked to APOE overexpression [39]. Moreover, APOE is demonstrated to be a strong genetic risk factor for AD and LBD [40]. Our study showed that APOE played a major role in ERBB4 signaling and lipid metabolism. Dysregulated lipid metabolism is demonstrated to be a risk factor for CRC and LBD [40,41]. Several malignancies, including CRC, are associated with the mutation or increased expression of ERBB4 [42]. ERBB4 promotes the progression of CRC by promoting epithelial-mesenchymal transition [43]. Research showed that ERBB4 played an essential role in AD [44]. However, research on the association between ERBB4 and LBD is limited. Our findings indicated that ERBB4 signaling and lipid metabolism seemed likely to play a role in the LBD-colon cancer pathway. We provided a new perspective on the association between LBD and colon cancer. Further research is needed to explore potential underlying mechanisms between these two diseases.

There are several major strengths in the study. First, the main strength was the MR design, which was based on genetic association data, and the results were not influenced by confounding factors. Second, we provided an explanation of the possible mechanisms underlying these relationships together with previous research, which strengthened the reliability of our findings. Third, we demonstrated that AD and LBD had causal effects on CRC. Therefore, it was necessary to strengthen the prevention and screening of CRC in patients with neurodegenerative diseases. Fourth, we conducted a bidirectional MR analysis to examine the direction of the causal relations.

However, our study has several limitations. First, the GWAS data we used for this research were collected from the European population. It means whether our results correspond in other populations remained confirmed. Second, although the GWAS data of neurodegenerative diseases and CRC came from different samples in our study, it is inevitable that there may be a sample overlap. Third, we used a more relaxed significance threshold between IVs and FTD for obtaining more SNPs, which probably increased the risk of violating the first assumption of MR analysis. However, the *F*-statistic of each SNP was over 10, indicating that every IV included was robust. Fourth, we found no causal association between the other neurodegenerative diseases (PD, ALS, and FTD) and CRC, including colon cancer and rectum cancer. However, we cannot exclude the possibility that the association was not detected due to insufficient sample size at present. The association between neurodegenerative diseases and CRC might become significant, along with summary data from larger sample sizes of GWAS in the future.

## 5 Conclusion

In summary, our study demonstrated that genetic vulnerabilities to AD nominally increased the overall risk of CRC and colon cancer. Genetically predicted LBD indicated an elevated risk of colon cancer. ERBB4 signaling and lipid metabolism may mediate the causal association between LBD and colon cancer. These findings demonstrated neurodegenerative diseases’ effect on CRC, which deserves further investigation.

## Funding

This research received no external funding.

## Competing interests

The authors declare no conflict of interest.

### Ethical approval

Review and/or approval by an ethics committee was not needed for this study because this study used the data from publicly available databases.

### Informed consent statement

Not applicable.

### Consent to participate

Not applicable.

### Consent for publication

Not applicable.

## Data availability statement

Only publicly available data were used in this study; the data sources and processing of these data are described in the Materials and Methods. Further inquiries can be directed to the corresponding author.

## CRediT authorship contribution statement

**Feifan Wang:** Writing – original draft, Software, Methodology, Conceptualization. **Lu Chen:** Validation, Methodology. **Mengke Nie:** Visualization, Validation. **Zhongxin Li:** Writing – review & editing, Supervision, Funding acquisition, Conceptualization.

## Declaration of competing interest

The authors declare that they have no known competing financial interests or personal relationships that could have appeared to influence the work reported in this paper.
