# Supplementary material for: Integrative analysis of causal associations between neurodegenerative diseases and colorectal cancer
Source: Heliyon. 2024 Jul 30;10(15):e35432. doi: 10.1016/j.heliyon.2024.e35432 (PMC11336615; doi:10.1016/j.heliyon.2024.e35432)
Supplement: Multimedia component 1 [file mmc1.pdf]

# Exploring potential causal associations between neurodegenerative diseases and colorectal cancer using Mendelian randomization and colocalization analysis

**Feifan Wang[1,†], Lu Chen[2,†], Mengke Nie[3], Zhongxin Li[1,*]**

1 Gastrointestinal Disease Diagnosis and Treatment Center, The First Hospital of Hebei Medical University, Shijiazhuang 050000, China.

2 Department of Medical Oncology and Radiation Sickness, Peking University Third Hospital, Beijing 100191, China.

3 Department of General Practice, Huaihe Hospital of Henan University, Kaifeng, 475000, China.

* Correspondence: Zhongxin Li, Doctor of Medicine, The First Hospital of Hebei Medical University, 89 Donggang Road, Shijiazhuang, 050000, China. Email: lizhongxin99@163.com.

† These authors contributed equally to this work.

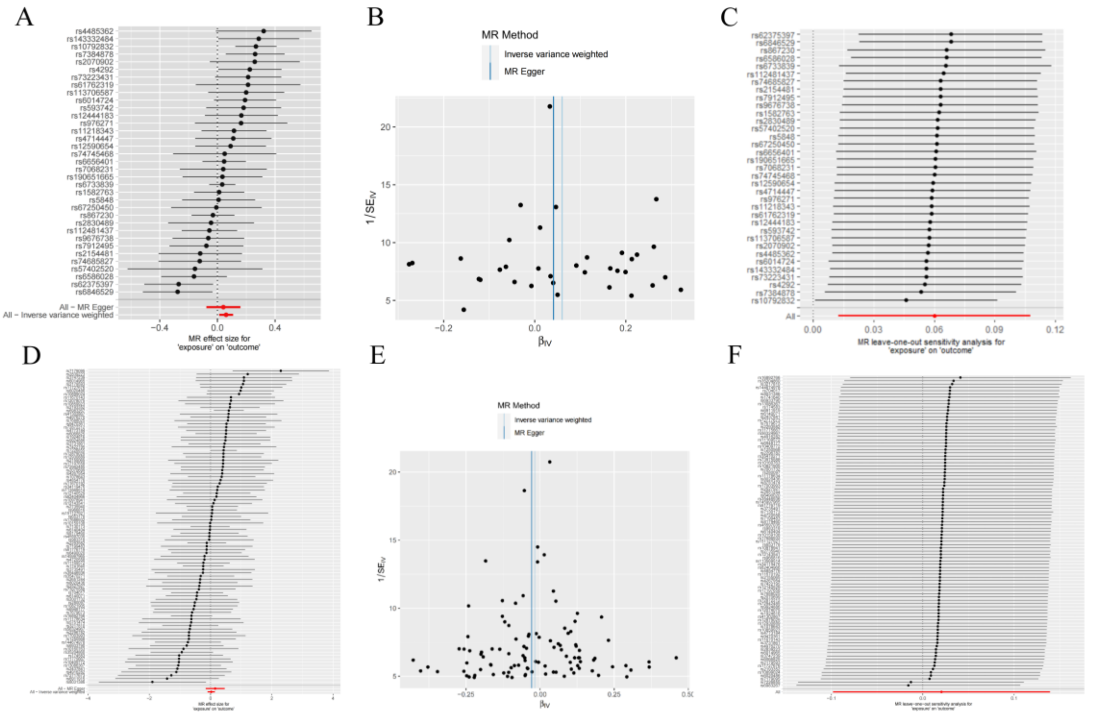


Figure S1 (A) Forest plot of AD on CRC for MR analysis. (B) Funnel plot of AD on CRC for MR analysis. (C) Leave-one-out sensitivity analysis plot of AD on CRC for MR analysis. (D) Forest plot of CRC on AD for MR analysis. (E) Funnel plot of CRC on AD for MR analysis. (F) Leave-one-out sensitivity analysis plot of CRC on AD for MR analysis.

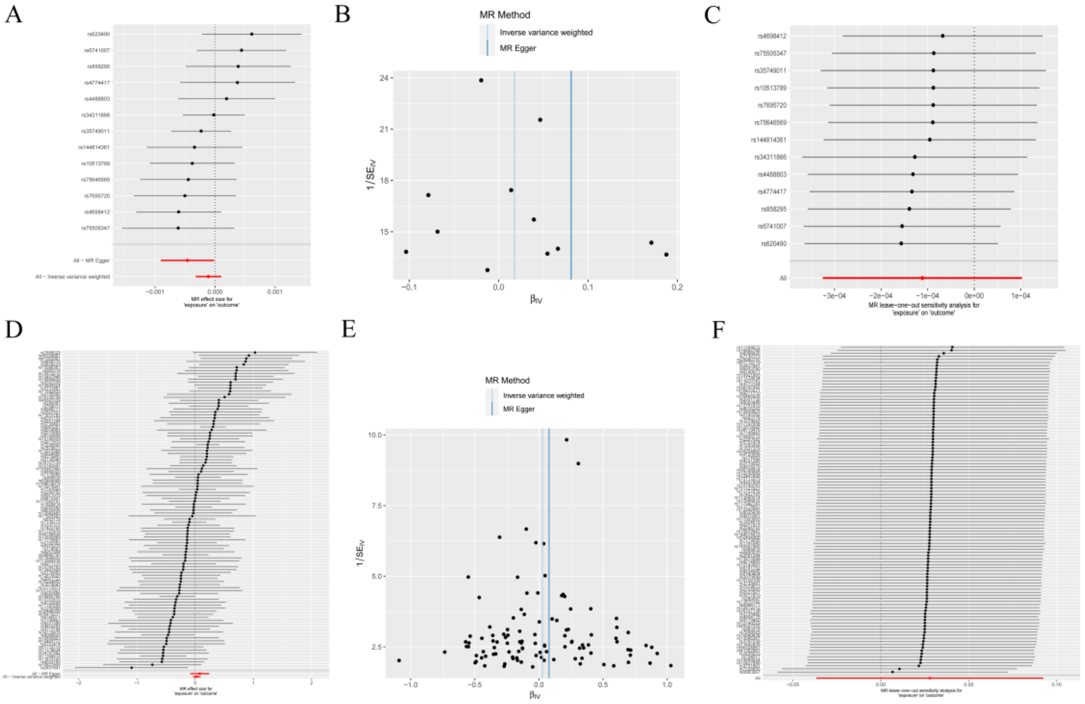


Figure S2 (A) Forest plot of PD on CRC for MR analysis. (B) Funnel plot of PD on CRC for MR analysis. (C) Leave-one-out sensitivity analysis plot of PD on CRC for MR analysis. (D) Forest plot of CRC on PD for MR analysis. (E) Funnel plot of CRC on PD for MR analysis. (F) Leave-one-out sensitivity analysis plot of CRC on PD for MR analysis.

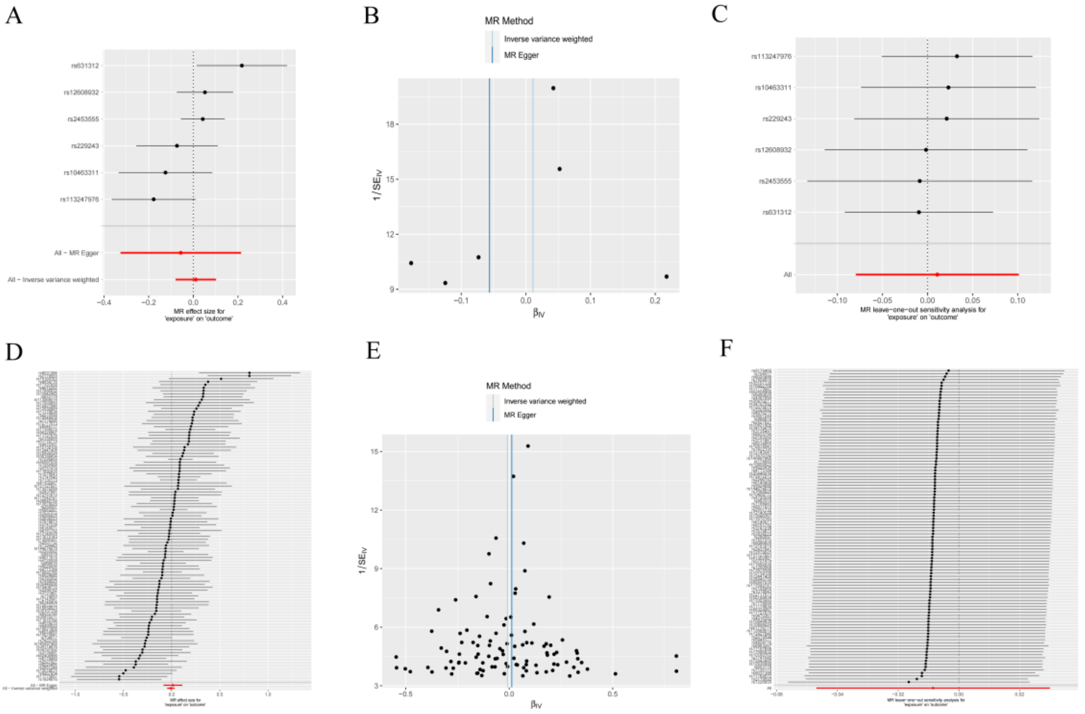


Figure S3 (A) Forest plot of ALS on CRC for MR analysis. (B) Funnel plot of ALS on CRC for MR analysis. (C) Leave-one-out sensitivity analysis plot of ALS on CRC for MR analysis. (D) Forest plot of CRC on ALS for MR analysis. (E) Funnel plot of CRC on ALS for MR

analysis. (F) Leave-one-out sensitivity analysis plot of CRC on ALS for MR analysis.

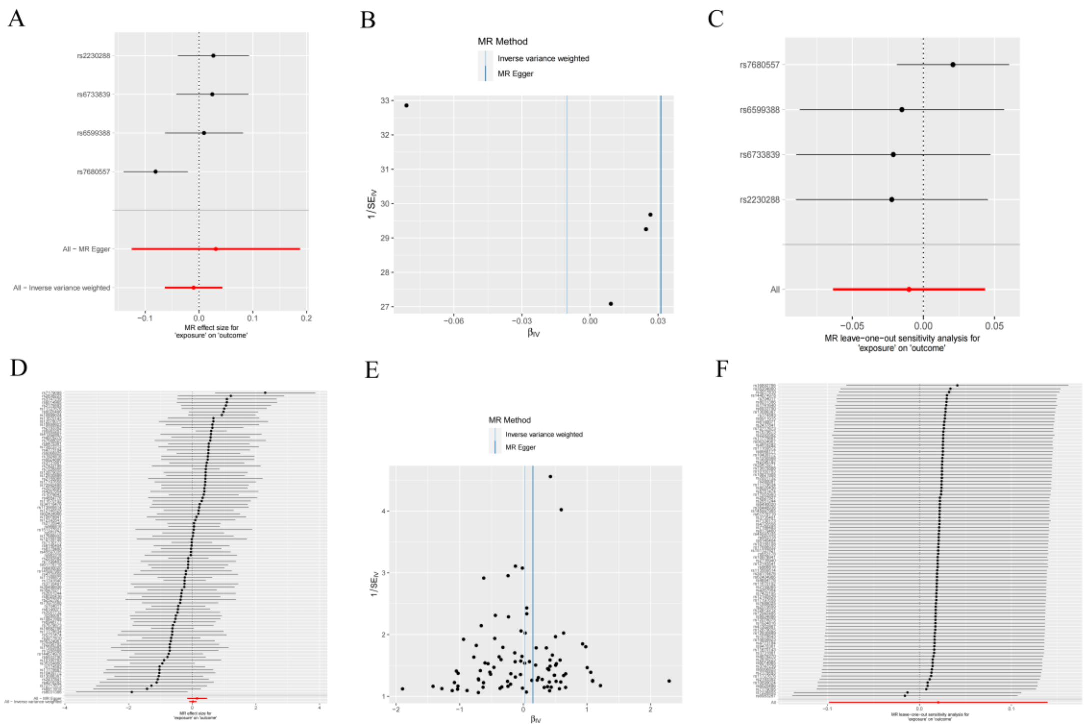


Figure S4 (A) Forest plot of LBD on CRC for MR analysis. (B) Funnel plot of LBD on CRC for MR analysis. (C) Leave-one-out sensitivity analysis plot of LBD on CRC for MR analysis. (D) Forest plot of CRC on LBD for MR analysis. (E) Funnel plot of CRC on LBD for MR analysis. (F) Leave-one-out sensitivity analysis plot of CRC on LBD for MR analysis.

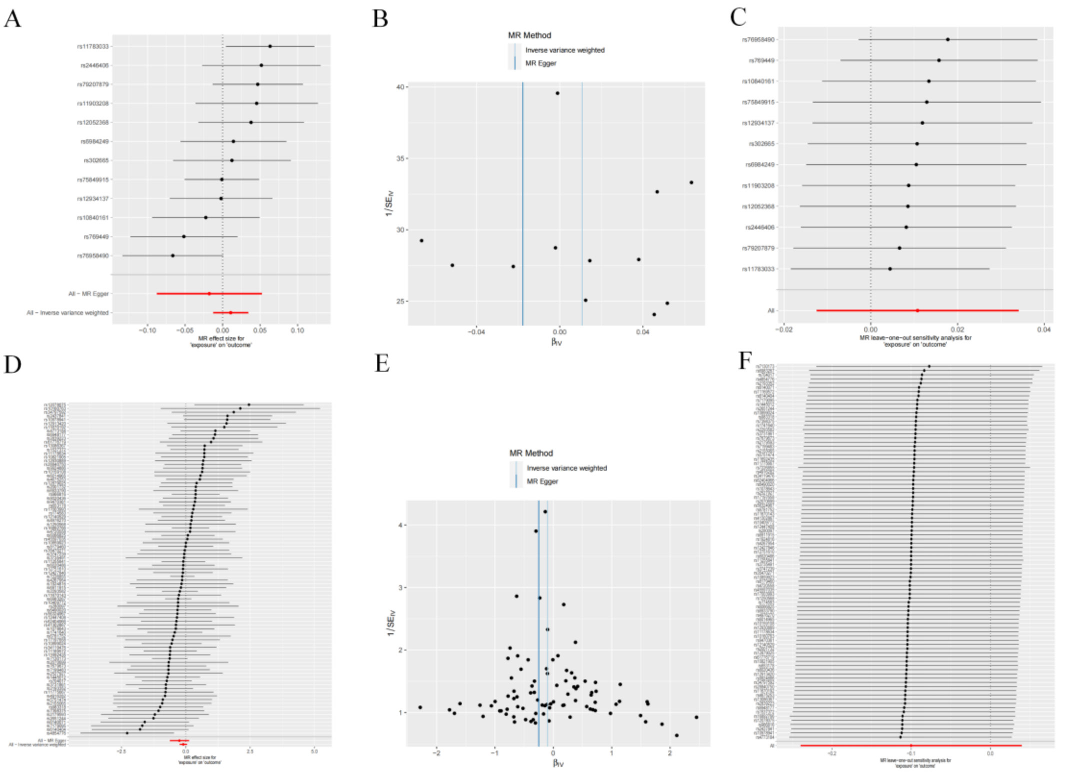


Figure S5 (A) Forest plot of FTD on CRC for MR analysis. (B) Funnel plot of FTD on CRC

for MR analysis. (C) Leave-one-out sensitivity analysis plot of FTD on CRC for MR analysis. (D) Forest plot of CRC on FTD for MR analysis. (E) Funnel plot of CRC on FTD for MR analysis. (F) Leave-one-out sensitivity analysis plot of CRC on FTD for MR analysis.

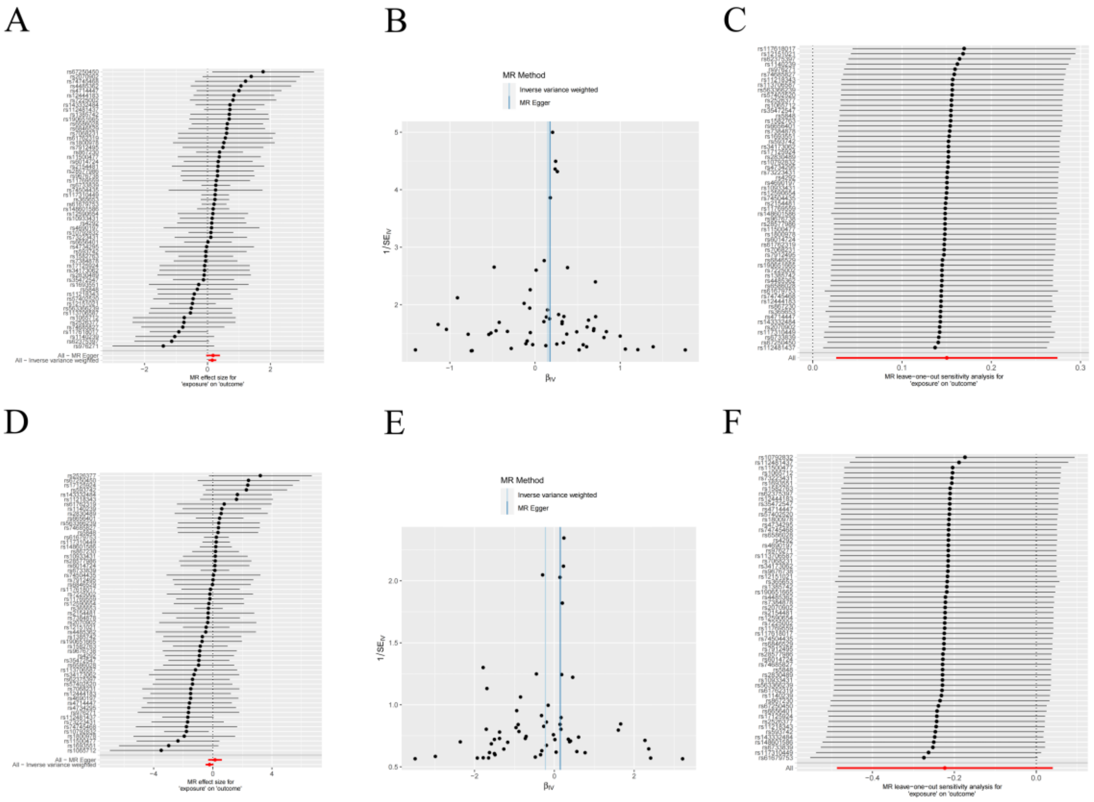


Figure S6 (A) Forest plot of AD on colon cancer for MR analysis. (B) Funnel plot of AD on colon cancer for MR analysis. (C) Leave-one-out sensitivity analysis plot of AD on colon cancer for MR analysis. (D) Forest plot of AD on rectum cancer for MR analysis. (E) Funnel plot of AD on rectum cancer for MR analysis. (F) Leave-one-out sensitivity analysis plot of AD on rectum cancer for MR analysis.

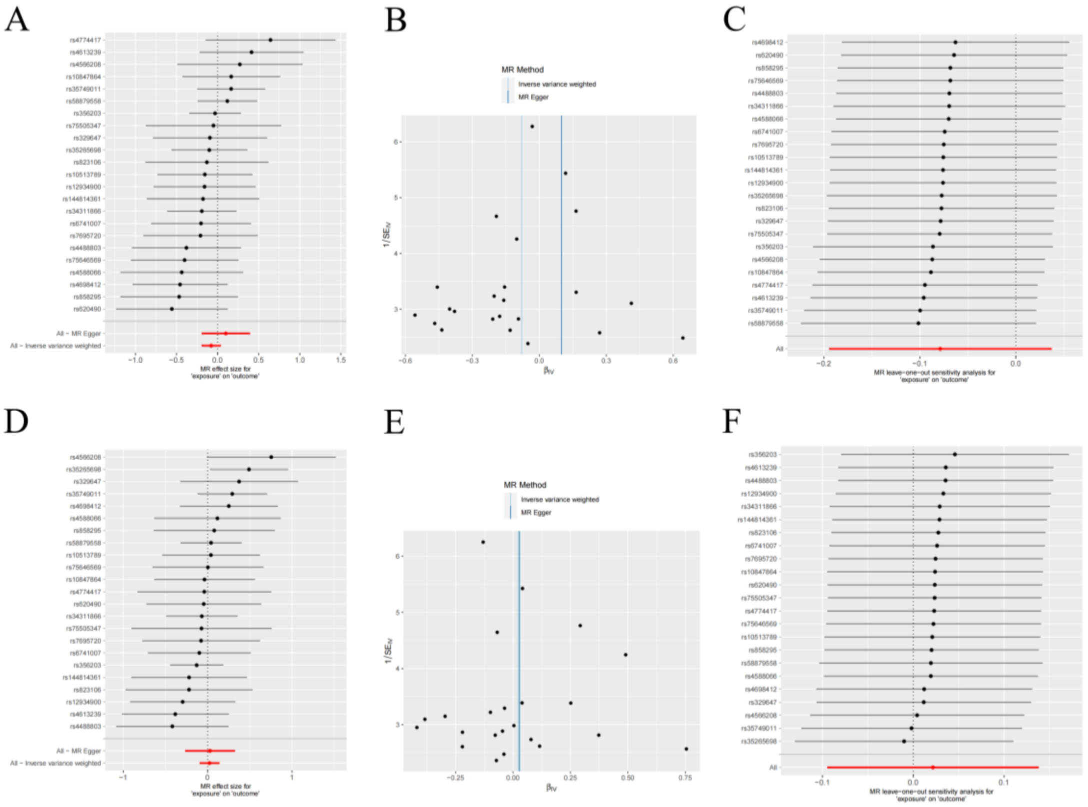


Figure S7 (A) Forest plot of PD on colon cancer for MR analysis. (B) Funnel plot of PD on colon cancer for MR analysis. (C) Leave-one-out sensitivity analysis plot of PD on colon cancer for MR analysis. (D) Forest plot of PD on rectum cancer for MR analysis. (E) Funnel plot of PD on rectum cancer for MR analysis. (F) Leave-one-out sensitivity analysis plot of PD on rectum cancer for MR analysis.

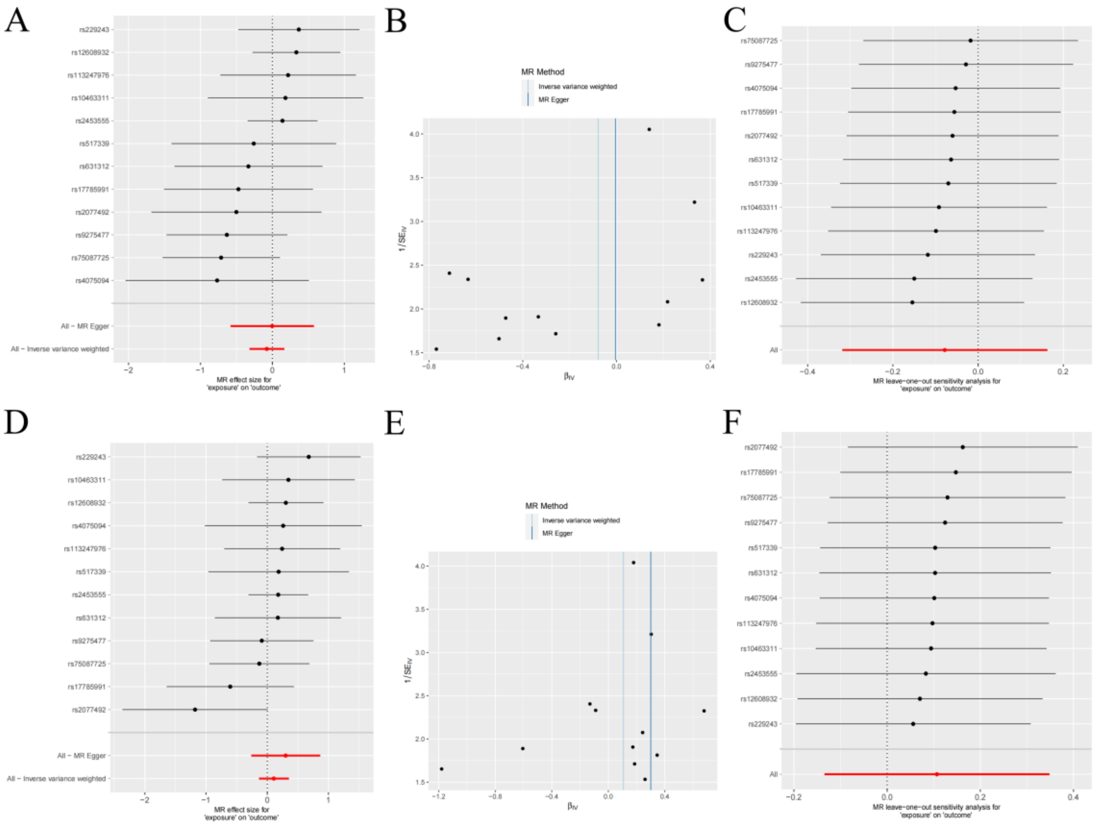


Figure S8 (A) Forest plot of ALS on colon cancer for MR analysis. (B) Funnel plot of ALS on colon cancer for MR analysis. (C) Leave-one-out sensitivity analysis plot of ALS on colon cancer for MR analysis. (D) Forest plot of ALS on rectum cancer for MR analysis. (E) Funnel plot of ALS on rectum cancer for MR analysis. (F) Leave-one-out sensitivity analysis plot of ALS on rectum cancer for MR analysis.

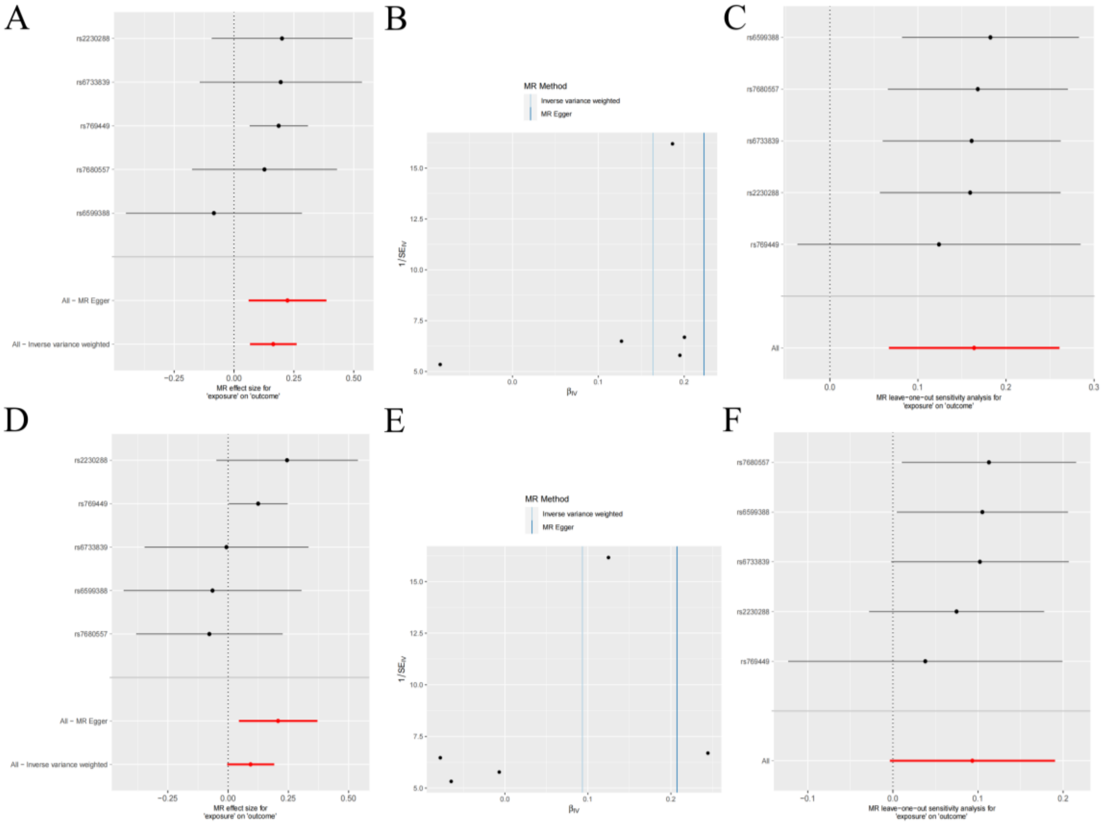


Figure S9 (A) Forest plot of LBD on colon cancer for MR analysis. (B) Funnel plot of LBD on colon cancer for MR analysis. (C) Leave-one-out sensitivity analysis plot of LBD on colon cancer for MR analysis. (D) Forest plot of LBD on rectum cancer for MR analysis. (E) Funnel plot of LBD on rectum cancer for MR analysis. (F) Leave-one-out sensitivity analysis plot of LBD on rectum cancer for MR analysis.

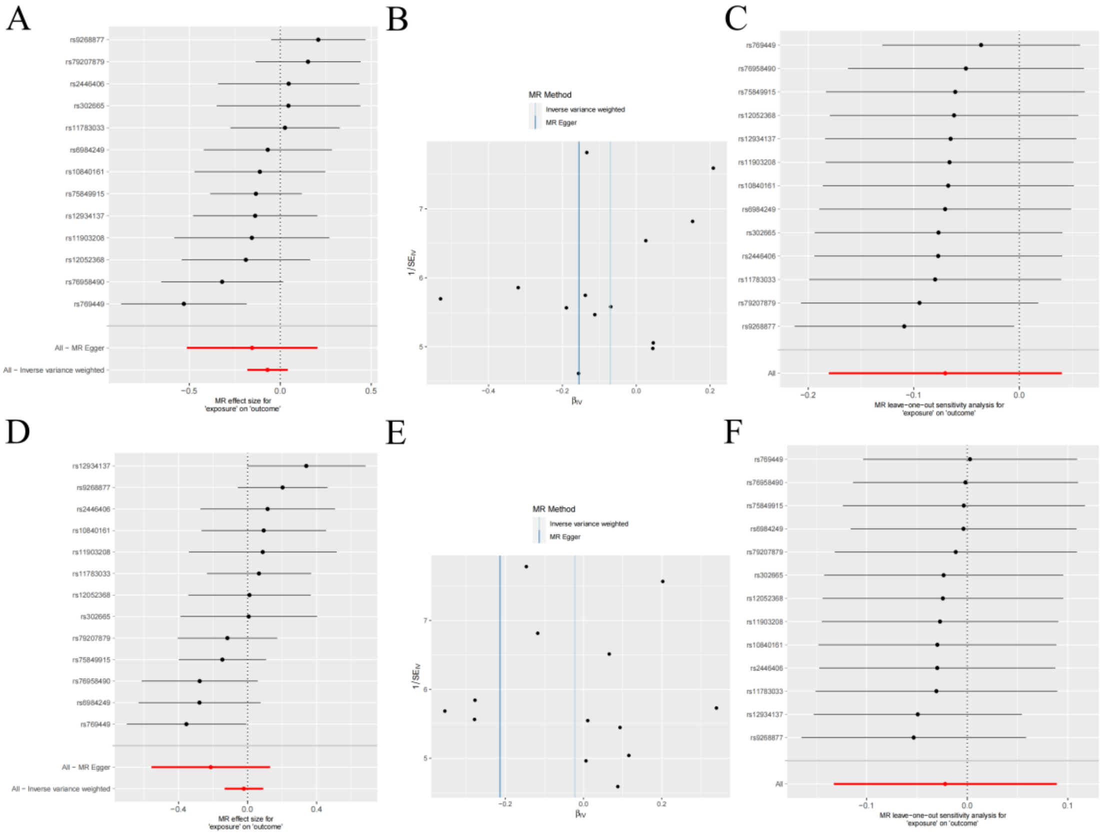


Figure S10 (A) Forest plot of FTD on colon cancer for MR analysis. (B) Funnel plot of FTD on colon cancer for MR analysis. (C) Leave-one-out sensitivity analysis plot of FTD on colon cancer for MR analysis. (D) Forest plot of FTD on rectum cancer for MR analysis. (E) Funnel plot of FTD on rectum cancer for MR analysis. (F) Leave-one-out sensitivity analysis plot of FTD on rectum cancer for MR analysis.

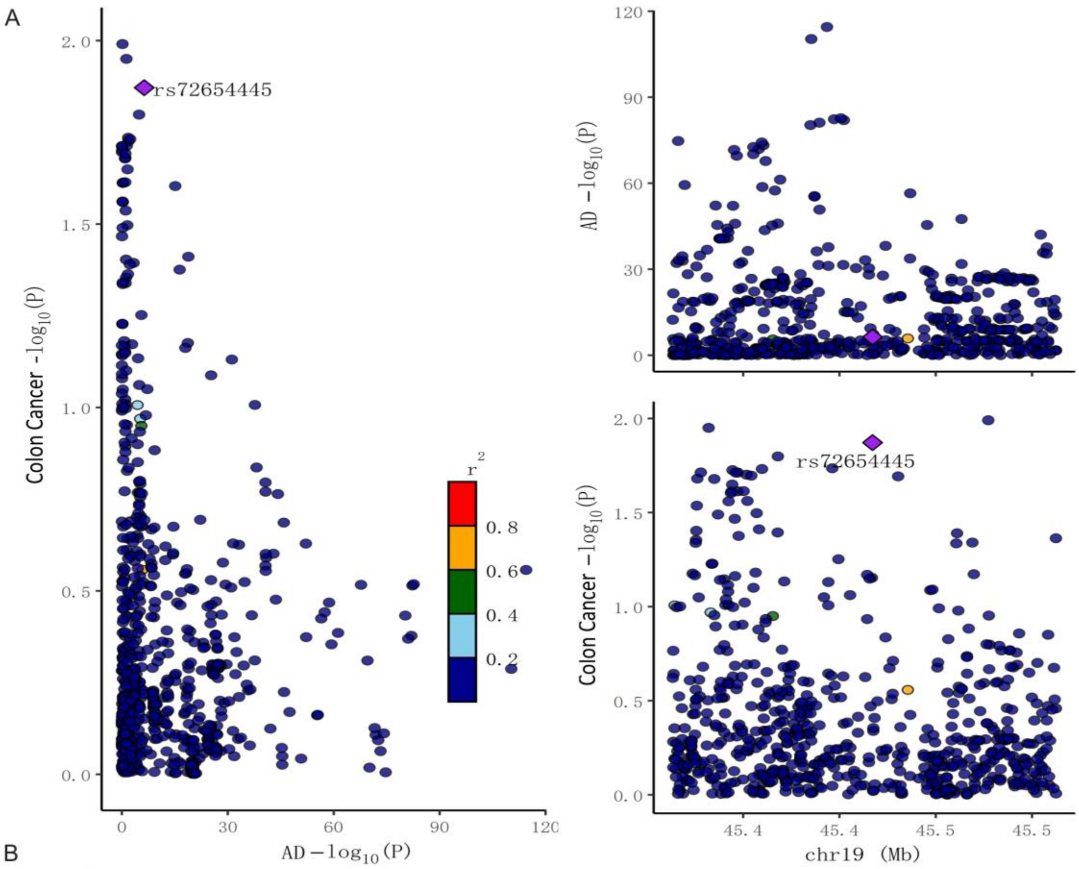


B

| No. of SNPs | PP.H0 | PP.H1 | PP.H2 | PP.H3 | PP.H4 |
|---|---|---|---|---|---|
| 734 | 9.12E-108 | 87.4% | 4.69E-109 | 4.5% | 8.1% |

Figure S11. (A) Regional association plots for AD and colon cancer at the chromosome 19 locus overlapping APOC1. (B) Colocalization analysis of AD and colon cancer. PP.H0 = neither AD nor colon cancer risk has a genetic association in the region, PP.H1 = only AD has a genetic association in the region, PP.H2 = only colon cancer risk has a genetic association in the region, PP.H3 = both AD and colon cancer risk are associated but have different causal variants, PP.H4= both AD and colon cancer risk are associated and share a single causal variant.
